# Association between hypomagnesemia and coagulopathy in sepsis: a retrospective observational study

**DOI:** 10.1186/s12871-022-01903-2

**Published:** 2022-11-24

**Authors:** Ken Tonai, Shinshu Katayama, Kansuke Koyama, Naho Sata, Yoshihiro Tomioka, Hisashi Imahase, Shin Nunomiya

**Affiliations:** Division of Intensive Care, Department of Anesthesiology and Intensive Care Medicine, Jichi Medical University School of Medicine, 3311-1, Yakushiji, Shimotsuke, Tochigi, 329-0498 Japan

**Keywords:** Magnesium, Hypomagnesemia, Sepsis, Disseminated intravascular coagulation, Coagulopathy

## Abstract

**Background:**

Hypomagnesemia reportedly has significant associations with poor clinical outcomes such as increased mortality and septic shock in patients with sepsis. Although the mechanism underlying these outcomes mostly remains unclear, some experimental data suggest that magnesium deficiency could potentiate coagulation activation in sepsis. However, in sepsis, the association between serum magnesium levels and coagulopathy, including disseminated intravascular coagulation (DIC), remains unknown. Thus, we aimed to investigate the relationship between serum magnesium levels and coagulation status and the association between hypomagnesemia and DIC in patients with sepsis.

**Methods:**

This retrospective observational study was conducted at the intensive care unit (ICU) of a university hospital from June 2011 to December 2017. Patients older than 19 years who met the Sepsis-3 definition were included. We categorized patients into three groups according to their serum magnesium levels: hypomagnesemia (< 1.6 mg/dL), normal serum magnesium level (1.6–2.4 mg/dL), and hypermagnesemia (> 2.4 mg/dL). We investigated the association between serum magnesium levels and overt DIC at the time of ICU admission according to the criteria of the International Society on Thrombosis and Haemostasis.

**Results:**

Among 753 patients included in this study, 181 had DIC, 105 had hypomagnesemia, 552 had normal serum magnesium levels, and 96 had hypermagnesemia. Patients with hypomagnesemia had a more activated coagulation status indicated by lower platelet counts, lower fibrinogen levels, higher prothrombin time-international normalized ratios, higher thrombin-antithrombin complex, and more frequent DIC than those with normal serum magnesium levels and hypermagnesemia (DIC: 41.9% vs. 20.6% vs. 24.0%, *P* < 0.001). The coagulation status in patients with hypomagnesemia was more augmented toward suppressed fibrinolysis than that in patients with normal serum magnesium levels and hypermagnesemia. Multivariate logistic regression revealed that hypomagnesemia was independently associated with DIC (odds ratio, 1.69; 95% confidence interval, 1.00–2.84; *P* = 0.048) after adjusting for several confounding variables.

**Conclusions:**

Patients with hypomagnesemia had a significantly activated coagulation status and suppressed fibrinolysis. Hypomagnesemia was independently associated with DIC in patients with sepsis. Therefore, the treatment of hypomagnesemia may be a potential therapeutic strategy for the treatment of coagulopathy in sepsis.

**Supplementary Information:**

The online version contains supplementary material available at 10.1186/s12871-022-01903-2.

## Background

Magnesium is an abundant intracellular cation that plays a role as a cofactor in many enzymatic reactions and is involved in several crucial biochemical processes such as oxidative metabolism, protein and nucleic acid synthesis, and immune responses [1]. Hypomagnesemia has frequently been identified in critically ill patients [2], and magnesium deficiency has been implicated in the pathophysiology of cardiovascular dysfunction, neuromuscular diseases, central nervous system dysfunction, and electrolyte disturbances [1, 3]. Meta-analysis has also shown a significant association between hypomagnesemia in critically ill patients and increased mortality, the need for mechanical ventilation, occurrence of sepsis, and prolonged stays in the intensive care unit (ICU) [4].

Sepsis is defined as life-threatening organ dysfunction caused by a dysregulated host response to infection [5]. Magnesium reportedly has immunomodulatory effects and is associated with dysregulated host response to infection and the pathophysiology of sepsis [6, 7]. Previous studies reported that hypomagnesemia was associated with lactic acidosis in sepsis [8], increased incidence of sepsis or septic shock in critically ill patients [9, 10], and increased mortality in sepsis [11]. However, the association between serum magnesium and the pathophysiology of sepsis mostly remains unclear.

We focused the association between serum magnesium levels and the pathophysiology of disseminated intravascular coagulation (DIC) in sepsis because the latter is one of the crucial complications of sepsis that causes microvascular thrombosis leading to organ failure and increased mortality [12]. Magnesium deficiency in an experimental sepsis model promotes the secretion of inflammatory cytokines [13] and lethal mediators of sepsis [14]. These cytokines and mediators dysregulate the immune responses, promote tissue damage, and activate coagulation via crosstalk between inflammation and coagulation [15]. Furthermore, treatment of DIC may be a potential therapeutic strategy for the treatment of sepsis because anticoagulant therapy in sepsis reduced mortality in patients with DIC, but not in those without DIC [16]. Therefore, if magnesium metabolism is associated with DIC, then the treatment of magnesium metabolism abnormalities in sepsis may be a potential therapeutic strategy for the treatment of DIC. However, no study has reported an association between magnesium levels and DIC at the time of ICU admission in patients with sepsis.

We hypothesized that hypomagnesemia is associated with DIC in patients with sepsis. Thus, we aimed to investigate the relationship between different serum magnesium levels and coagulation status, and the association between hypomagnesemia and DIC upon ICU admission in patients with sepsis.

## Methods

### Study design

This was a single-center observational retrospective study in the 16-bed ICU of Jichi Medical University Hospital, Tochigi, Japan. Data were extracted from electronic medical records. Patients admitted with sepsis between June 2011 and December 2017 were included in the study if they were older than 19 years and met the Sepsis-3 definition [5]. Patients readmitted to the ICU, or those who had no data on the parameters included in the multivariate logistic regressions, such as serum magnesium concentration and overt DIC (according to the International Society on Thrombosis and Haemostasis [ISTH]) [17], were excluded from this study. This study was approved by the ethics committee of Jichi Medical University Hospital (21-034). All patients included in this study were provided with the details of this study and the opportunity to opt out on the website of Jichi Medical University Hospital. This method was approved by Jichi Medical University Hospital Bioethics Committee for Clinical Research and did not require that informed consent be provided because of the retrospective observational nature.

### Data collection

At our institute, we routinely measure global biomarkers that are associated with organ failure and coagulation biomarkers in patients with sepsis at the time of admission to the ICU. We retrospectively collected the following variables: age, sex, body mass index, origin of sepsis, medical history (anticoagulation therapy, chronic kidney disease with dialysis, diabetes mellitus, chronic liver disease, and hypertension), blood cell count, serum biomarkers (albumin, bilirubin, creatinine, C-reactive protein [CRP], ionized calcium, lactate, and magnesium), plasma biomarkers (prothrombin time, prothrombin time-international normalized ratio [PT-INR], fibrinogen, fibrin degradation products [FDP], antithrombin [AT] III activity, protein C [PC] activity, thrombin-antithrombin complex [TAT], plasmin-α2 plasmin inhibitor [PIC], and plasminogen activator inhibitor-1 [PAI-1]), Acute Physiology and Chronic Health Evaluation (APACHE) II score, Sequential Organ Failure Assessment score, PaO_2_/FIO_2_ ratio, presence of septic shock, mechanical ventilation support, renal replacement therapy, DIC, and in-hospital death.

### Definitions of serum magnesium level and DIC

Serum magnesium concentration was categorized into three groups according to the definition from previous studies [18–20]: hypomagnesemia (< 1.6 mg/dL), normal magnesium level (1.6–2.4 mg/dL), and hypermagnesemia (> 2.4 mg/dL). DIC was diagnosed based on the overt DIC criteria of the ISTH [17]. The ISTH overt DIC score was calculated using platelet count (1 point: ≥ 50,000 to < 100,000/μL; 2 points: < 50,000/μL), FDP level (2 points: moderate increase [≥ 10 to < 25 μg/mL]; 3 points: strong increase [≥ 25 μg/mL]), prolongation of prothrombin time (1 point: ≥ 3 to < 6 seconds; 2 points: ≥ 6 seconds), and fibrinogen level (1 point: < 100 mg/dL). If the sum of points was more than 4, the patient was diagnosed as having overt DIC.

### Biomarker measurement

Global markers (platelet count, fibrinogen, PT-INR, and FDP) were assayed using an XE-5000 hematology analyzer (Sysmex, Kobe, Japan) and a CS-2100i automatic coagulation analyzer (Sysmex, Kobe, Japan). AT III and PC activity (Berichrom assays [Siemens Healthcare Diagnostics, Tokyo, Japan]), TAT and PIC (TAT/PIC test F enzyme immunoassay [Sysmex, Kobe, Japan]), and PAI-1 (tPAI test [Mitsubishi Chemical Medience, Tokyo, Japan]) were measured with commercially available assay kits.

### Statistical analyses

Continuous variables are presented as means and standard deviations, or median values and interquartile ranges depending on the type of distribution. Categorical variables are presented as frequencies and percentages. Comparisons between the groups for continuous variables were made using the Mann–Whitney *U* test and Kruskal-Wallis test. Categorical variables were compared using the chi-square test between the DIC and non-DIC groups, as well as between the two or three groups according to serum magnesium levels. The Steel–Dwass test and chi-square test with Bonferroni correction were applied to determine significance in the setting of multiple comparisons between the three groups according to serum magnesium levels or the quantile of serum ionized calcium. Univariate and multivariate logistic regression analyses were performed to determine the independent associations for DIC in complete-case analyses. Taking into consideration collinearity and the number of patients who experienced the outcome of interest, variables that were considered to affect DIC or sepsis-induced coagulopathy in previous studies such as sex, APACHE II score, and laboratory data on admission (magnesium levels, bilirubin, creatinine, CRP, ionized calcium, and lactate) [21–24], were included in the multivariate logistic regression model. We constructed receiver operating characteristic (ROC) curves and measured the areas under the curves (AUC) with 95% confidence intervals (CI) to evaluate the predictive validity of the serum magnesium concentration for DIC. Sensitivity analyses were conducted in subgroup divided by the site of origin of sepsis (abdomen or non-abdomen) to assess the consistency of the association between serum magnesium levels and DIC. All analyses were performed using the JMP® 13 software program (SAS Institute Inc., Cary, NC, USA) and R package (version 4.0.4). Statistical significance was set at *P* < 0.05.

## Results

### Patient characteristics

**Fig. 1:**
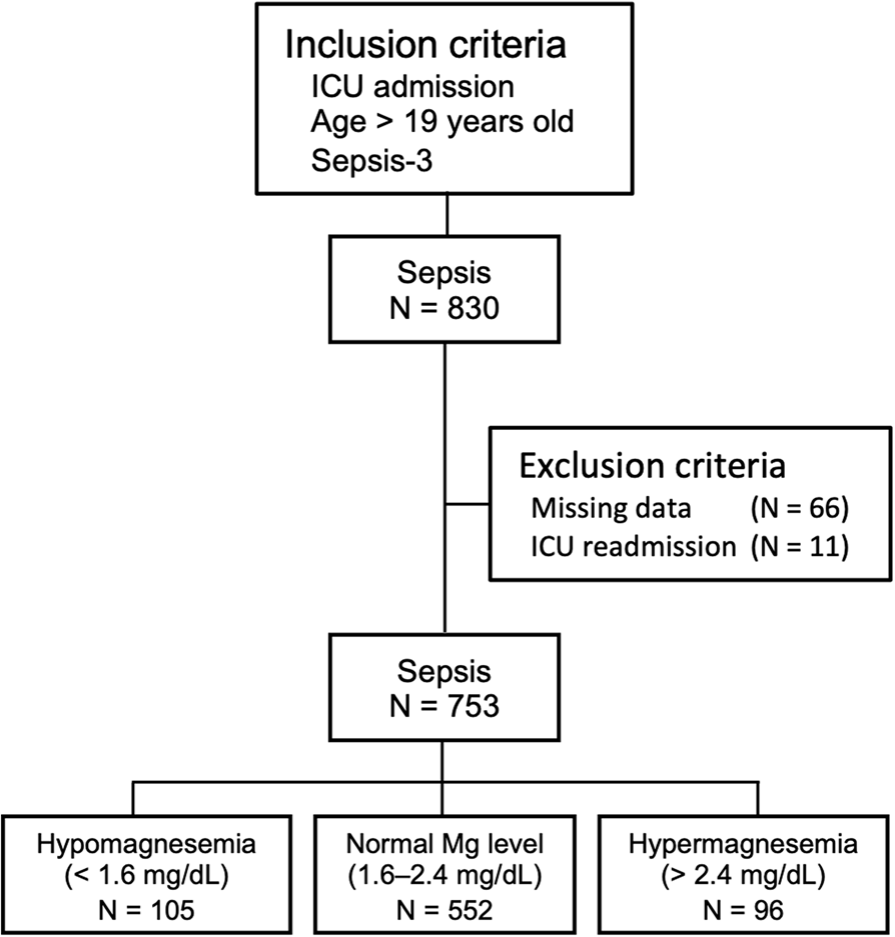
Flowchart describing the study population

From June 2011 to December 2017, a total of 830 patients with sepsis were admitted to the ICU of Jichi Medical University Hospital. Eleven patients who were readmitted to the ICU were excluded from the study. Sixty-six patients with missing data at the time of ICU admission included in multivariate logistic regressions were also excluded. A total of 753 patients were included in the data analysis. Based on the serum magnesium concentration at the time of ICU admission, 105, 552, and 96 patients were diagnosed as having hypomagnesemia (Mg < 1.6 mg/dL), normal magnesium level (Mg 1.6–2.4 mg/dL), and hypermagnesemia (Mg > 2.4 mg/dL), respectively (Fig. 1). Table 1 shows the baseline characteristics and comparisons between hypomagnesemia, normal magnesium level, and hypermagnesemia groups. Patients with hypomagnesemia had higher severity (SOFA score and APACHE II score), more frequent septic shock and DIC (41.9% vs. 20.7%; *P* < 0.05), and higher hospital mortality (25.7% vs. 14.1%; *P* < 0.05) than those with normal magnesium level. In contrast, patients with hypomagnesemia had more frequent septic shock and DIC (41.9% vs. 24.0%; *P* < 0.05) than those with hypermagnesemia although there were no differences in severity and hospital mortality between two groups.

**Table 1:** Patient characteristics and laboratory parameters of the study population

	Hypomagnesemia (< 1.6 mg/dL) *N* = 105	Normal Mg level (1.6–2.4 mg/dL) *N* = 552	Hypermagnesemia (> 2.4 mg/dL) *N* = 96	*P*-value
Age, years, median (IQR)	65 (53–74)	68 (59–78) *	70 (63–78) *	0.024
Male sex, n (%)	53 (50.5)	312 (56.5)	51 (53.1)	0.47
BMI	23.0 (20.2–25.4)	22.0 (19.4–25.3)	23.3 (19.5–25.6)	0.20
Medical history, n (%)				
Anticoagulation therapy	8 (7.6)	34 (6.2)	14 (14.6) †	0.015
CKD with dialysis	2 (1.9)	43 (7.8)	11 (11.5) *	0.030
Diabetes mellitus	21 (20.0)	157 (28.4)	32 (33.3)	0.093
Chronic liver disease	15 (14.3)	47 (8.5)	8 (8.3)	0.16
Hypertension	41 (39.1)	281 (50.9)	57 (59.4) *	0.014
Origin of sepsis, n (%)				0.12
Abdomen	46 (43.8)	308 (55.8)	41 (42.7)	
Thorax	24 (22.9)	112 (20.3)	25 (26.0)	
Urinary tract	6 (5.7)	23 (4.2)	4 (4.2)	
Other	29 (27.6)	109 (19.8)	26 (27.1)	
Laboratory values, median (IQR)				
Magnesium, mg/dL	1.4 (1.3–1.5)	2.0 (1.8–2.2) *	2.7 (2.5–2.9) * †	< 0.001
Albumin, mg/dL	2.4 (1.9–2.9)	2.4 (2.0–2.8)	2.3 (2.1–2.8)	0.84
Bilirubin, mg/dL	0.92 (0.64–1.57)	0.85 (0.59–1.40)	0.72 (0.50–1.29) *	0.049
Creatinine, mg/dL	1.51 (0.83–2.20)	1.00 (0.67–1.96) *	1.72 (0.93–3.82) †	< 0.001
CRP, mg/dL	9.5 (5.0–16.0)	13.7 (5.9–23.5) *	13.4 (7.7–21.1) *	0.019
Ionized calcium, mmol/L	0.99 (0.92–1.07)	1.06 (1.0–1.11) *	1.06 (0.98–1.16) *	< 0.001
Lactate, mmol/L	3.9 (2.2–6.1)	2.1 (1.3–3.3) *	2.1 (1.4–3.6) *	< 0.001
Organ support, n (%)				
Mechanical ventilation	91 (86.7)	453 (82.1)	81 (84.4)	0.48
Renal replacement therapy	34 (32.4)	112 (20.3) *	35 (36.5) †	< 0.001
Severity of disease				
SOFA score on ICU admission, median (IQR)	9 (6–11)	7 (4–9) *	9 (6–11) †	< 0.001
APACHE II score, median (IQR)	25 (20–33)	23 (17–29) *	27 (20–33) †	< 0.001
Septic shock, n (%)	74 (70.1)	233 (42.3) *	44 (45.8) *	< 0.001
PaO_2_/F_I_O_2_ ratio, median (IQR)	259 (155–340)	268 (173–363)	237 (155–360)	0.29
ISTH score, median (IQR)	4 (3–5)	3 (2–4) *	3 (2–4) *	< 0.001
DIC, n (%)	44 (41.9)	114 (20.7) *	23 (24.0) *	< 0.001
Hospital mortality, n (%)	27 (25.7)	78 (14.1) *	24 (25.0) †	0.002

Continuous variables are presented as medians with interquartile ranges (first to third quartiles). Categorical variables are presented as counts and percentiles

Abbreviations: *Mg* magnesium, *IQR* interquartile range (first quartile to third quartile), *BMI* body mass index, *CKD* chronic kidney disease, *CRP* C-reactive protein, *SOFA* Sequential Organ Failure Assessment, *APACHE* Acute Physiology and Chronic Health Evaluation, *PaO2* partial pressure of oxygen, *FIO2* fraction of inspired oxygen, *ISTH* International Society on Thrombosis and Hemostasis, *DIC* disseminated intravascular coagulation.

*P-value < 0.05, comparison versus hypomagnesemia (Steel–Dwass test or the chi-square test with Bonferroni correction)

†*P*-value < 0.05, comparison versus normal level (Steel–Dwass test or the chi-square test with Bonferroni correction)

According to the ISTH criteria, 181 patients had overt DIC. Patients with DIC had higher levels of bilirubin, creatinine, and lactate than those without DIC. Compared to the group without DIC, the DIC group had a higher severity of disease: higher APACHE II score (median: 29 vs 23; *P* < 0.001) and higher hospital mortality (33.7% vs. 11.9%; *P* < 0.001) (Additional file 1). The median serum magnesium concentration at the time of ICU admission was 1.9 mg/dL and 2.0 mg/dL in those with DIC and without DIC, respectively (*P* = 0.007). However, the histogram of serum magnesium concentration showed a horizontal distribution of patients with DIC towards low values compared with those without DIC (Additional file 2). The frequency of DIC according to decile (sorted into ten equal parts) of serum magnesium concentration on ICU admission demonstrated a J-shaped curve (Additional file 2).

### Comparison of coagulation parameters according to serum magnesium levels on ICU admission

**Fig. 2:**
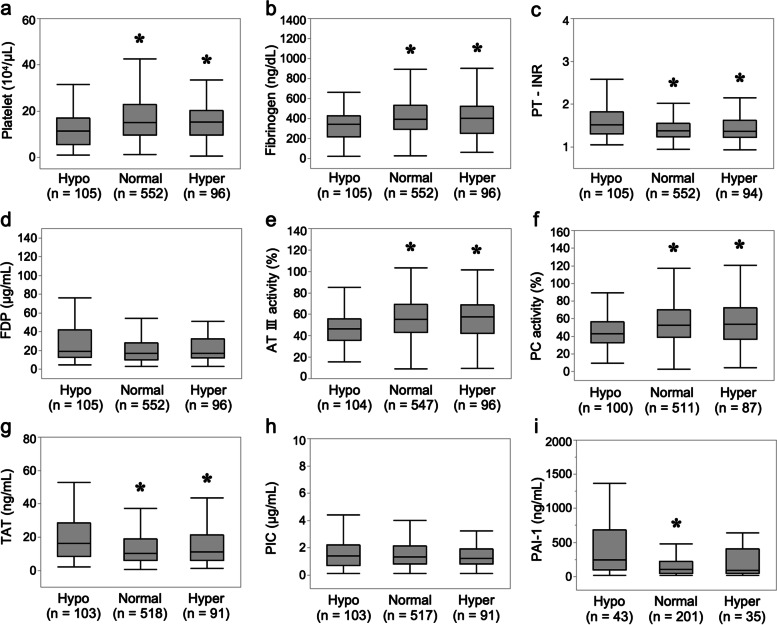
Coagulation parameters between different serum magnesium levels (hypomagnesemia, normal magnesium level, and hypermagnesemia) in sepsis. Box-and-whisker plot depicting a difference in coagulation parameters between different serum magnesium levels (hypomagnesemia [Hypo] vs. normal magnesium level [Normal] vs. hypermagnesemia [Hyper]) in patients with sepsis admitted to the ICU. Admission serum levels of **(a)** platelet count (10^4^/μL); **(b)** fibrinogen (mg/dL); **(c)** PT-INR; **(d)** FDP (μg/mL); **(e)** AT III activity (%); **(f)** PC activity (%); **(g)** TAT (ng/mL); **(h)** PIC (μg/mL); **(i)** PAI-1 (ng/mL). Boxplots display median with first and third quartile, and whiskers indicate smallest and largest nonoutlier observations. *P-value < 0.05, comparison versus hypomagnesemia (Steel–Dwass test). Abbreviations: AT III, antithrombin III; FDP, fibrin degradation products; ICU, intensive care unit; PC, protein C; PIC, plasmin-α2 plasmin inhibitor complex; PT-INR, prothrombin time-international normalized ratio; TAT, thrombin-antithrombin complex; PAI-1, plasminogen activator inhibitor-1

Compared to patients with normal magnesium levels and hypermagnesemia, those with hypomagnesemia had more activated coagulation status as follows: lower platelet count (median [10^4^/μL]: 11.2 vs. 14.9, *P* < 0.05; 11.2 vs. 15.1, *P* < 0.05, respectively), lower fibrinogen level (median [mg/dL]: 268 vs. 356, *P* < 0.05; 268 vs. 371, *P* < 0.05, respectively), higher PT-INR (median: 1.52 vs. 1.37, *P* < 0.05; 1.52 vs. 1.36, *P* < 0.05, respectively), and higher TAT (median [ng/mL]: 16.1 vs. 10.0, *P* < 0.05; 16.1 vs. 11.1, *P* < 0.05, respectively) in multiple comparisons. There were no differences in the marker of fibrinolysis: PIC and FDP between magnesium levels despite more activated coagulation markers in hypomagnesemia. Patients with hypomagnesemia also had a coagulation status of suppressed fibrinolysis. The marker of thrombin generation: TAT (median: 16.1 ng/mL) was markedly increased, the markers of anticoagulant activity: AT III activity (median: 46.2%) and PC activity (median: 42.6%) were moderately decreased, while the marker of fibrinolysis: PIC (median: 1.4 μg/mL) was mildly increased and the marker of suppressed fibrinolysis: PAI-1 (median: 242 ng/mL) was markedly increased. The statuses of activated coagulation and suppressed fibrinolysis were more deteriorated in patients with hypomagnesemia than in those with normal magnesium levels and hypermagnesemia (Fig. 2 and Additional file 3).

### Comparison of coagulation parameters according to serum magnesium levels on days 3 and 5

Based on the serum magnesium concentration on days 3 and 5; 62, 558, and 55 patients on day 3; and 30, 471, 41 patients on day 5 were categorized into hypomagnesemia (Mg < 1.6 mg/dL), normal magnesium level (Mg 1.6–2.4 mg/dL), and hypermagnesemia (Mg > 2.4 mg/dL), respectively. Patients with hypomagnesemia on day 3 had the statuses of activated coagulation and suppressed fibrinolysis as follows: lower platelet count, lower fibrinogen level, higher PT-INR, higher TAT, and lower PIC than those with normal magnesium levels and hypermagnesemia (Additional file 4). Although patients with hypomagnesemia on day 5 had lower platelet count and lower fibrinogen level, there were no significant differences in PT-INR, TAT, and PIC (Additional file 5). Patients with hypomagnesemia on day 3 had more frequent DIC from admission to day 3 (met the overt DIC criteria of the ISTH for more than a day by day 3) (35.5% vs. 26.7% vs. 20.8%; *P* = 0.013), and patients with hypomagnesemia on day 5 had no difference in the frequency of DIC from admission to day 5 (met the overt DIC criteria of the ISTH for more than a day by day 5) (60.0% vs. 42.0% vs. 46.3%; *P* = 0.14).

### Time course of coagulation parameters in persistent hypomagnesemia and resolved hypomagnesemia on day 3

Among 105 patients with hypomagnesemia (Mg < 1.6 mg/dL) on admission day, 99 patients were categorized into persistent hypomagnesemia group (Mg < 1.6 mg/dL on day 3) (N = 35) and resolved hypomagnesemia group (Mg ≧ 1.6 mg/dL on day 3) (N = 64) according to the serum magnesium concentration on day 3. There was no difference in the frequency of DIC from admission to day 3 (51.4% vs. 62.5%, *P* = 0.29), and coagulation parameters except for PAI-1 on days 3 and 5 between persistent hypomagnesemia and resolved hypomagnesemia (Additional file 6).

### Time course of coagulation parameters in developed hypomagnesemia and non-hypomagnesemia on day 3

Among 648 patients with non-hypomagnesemia (Mg ≧ 1.6 mg/dL) on ICU admission day, 576 patients were categorized into developed hypomagnesemia group (Mg < 1.6 mg/dL on day 3) (N = 27) and non-hypomagnesemia group (Mg ≧ 1.6 mg/dL on day 3) (N = 549) according to the serum magnesium concentration on day 3. Developed hypomagnesemia group had more frequent DIC from admission to day 3 (59.3% vs. 40.0%, *P* = 0.005), and had the statuses of activated coagulation and suppressed fibrinolysis on day 3 as follows: lower platelet count, lower fibrinogen level, higher PT-INR, higher TAT, and lower PIC than non-hypomagnesemia group (Additional file 7).

### Association between magnesium levels and DIC

Univariate logistic regression indicated that DIC was associated with hypomagnesemia (odds ratio [OR], 2.77; 95% CI, 1.79–4.30; *P* < 0.001) and the following factors: APACHE score (OR, 1.10 per score; 95% CI, 1.08–1.13; *P* < 0.001), bilirubin (OR, 1.12 per mg/dL; 95% CI, 1.05–1.20; *P* = 0.001), creatinine (OR, 1.14 per mg/dL; 95% CI, 1.06–1.22; *P* < 0.001), ionized calcium (OR, 0.008 per mmol/L; 95% CI, 0.001–0.046; *P* < 0.001), and lactate (OR, 1.38 per mmol/L; 95% CI, 1.28–1.48; *P* < 0.001). Multivariate logistic regression revealed an independent association between hypomagnesemia and DIC (OR, 1.69; 95% CI, 1.00–2.84; *P* = 0.048) (Table 2).

**Table 2:** Logistic regression analyses of the disseminated intravascular coagulation

	Univariate logistic regression *N* = 753			Multivariate logistic regression *N* = 753		
	OR	95% CI	*P*-value	OR	95% CI	*P-*value
Magnesium						
Normal Mg level	Reference			Reference		
Hypomagnesemia	2.77	1.79–4.30	< 0.001	1.69	1.00–2.84	0.048
Hypermagnesemia	1.21	0.73–2.02	0.46	0.82	0.44–1.50	0.51
Male	1.06	0.76–1.49	0.73	1.06	0.72–1.55	0.77
APACHE II score, per score	1.10	1.08–1.13	< 0.001	1.06	1.04–1.09	< 0.001
Bilirubin, per mg/dL	1.12	1.05–1.20	0.001	1.09	1.01–1.17	0.024
Creatinine, per mg/dL	1.14	1.06–1.22	< 0.001	1.05	0.95–1.15	0.37
CRP, per mg/dL	1.01	0.99–1.02	0.41	1.01	0.99–1.03	0.16
Ionized calcium, per mmol/L	0.008	0.001–0.046	< 0.001	0.094	0.013–0.75	0.020
Lactate, per mmol/L	1.38	1.28–1.48	< 0.001	1.26	1.17–1.36	< 0.001

Logistic regression analyses for disseminated intravascular coagulation.

Data are expressed as odds ratios (95% CI). Logistic regression analyses were performed for the complete-case analysis. A total of 753 participants were included in the multivariate logistic regression analyses. *Abbreviations*: *OR* odds ratio, *CI* confidence interval, *Mg* magnesium, *APACHE* Acute Physiology and Chronic Health Evaluation, CRP C-reactive protein

The predictive validity of serum magnesium concentration for DIC

The ROC analysis showed AUC of serum magnesium concentration was 0.57 (95% CI 0.51–0.62, sensitivity 30.4% and specificity 84.6% with a cut-off of 1.6mg/dL) (Additional file 8).

### The sensitivity analysis

Multivariate logistic regression revealed no independent association between hypomagnesemia and DIC among patients with abdominal sepsis (OR, 0.88; 95% CI, 0.39–2.02; *P* = 0.77), while an independent association was demonstrated between hypomagnesemia and DIC among patients with non-abdominal sepsis (OR, 3.04; 95% CI, 1.46–6.32; *P* = 0.003) (Additional file 9 and 10).

## Discussion

In this study, a comparison of serum magnesium levels revealed that hypomagnesemia was significantly associated with an activated coagulation and suppressed fibrinolysis status, as reflected by lower platelet counts and plasma fibrinogen levels, and higher PT-INR, TAT, and PAI-1 levels. We showed that hypomagnesemia was independently associated with coagulopathy defined as overt DIC after adjusting for several confounders. We also found that patients with hypomagnesemia had higher severity and in-hospital mortality than those with normal magnesium levels in sepsis as shown in a previous study [11].

We assessed which types of variables in serum magnesium were desirable to evaluate the association between serum magnesium and DIC. The histogram of DIC showed a horizontal distribution towards low values compared with the histogram of non-DIC despite relatively large amount of overlap including the peak between the two histograms. The frequency of DIC according to decile of serum magnesium concentration demonstrated a J-shaped curve (Additional file 2). Thus, we decided that it would be preferable to evaluate the association by serum magnesium concentration as a categorical variable rather than a continuous variable because of its non-linear relation with DIC. Serum magnesium concentration as a continuous variable has low predictive power as a biomarker for DIC, whereas we showed that hypomagnesemia as the categorical variable was associated with DIC.

In this study, we focused on whether hypomagnesemia could potentiate coagulopathy in sepsis. However, it remains unknown whether hypomagnesemia is associated with sepsis-induced coagulopathy. We investigated the coagulation profile in patients with hypomagnesemia from the perspective of DIC to clarify the type of coagulopathy associated with hypomagnesemia. Our data demonstrated that the coagulation profile in patients with DIC was suppressed-fibrinolytic-type DIC (Fig. 1 and Additional file 1), which was typical in sepsis-induced DIC as reflected by markedly increased TAT, mildly increased PIC, mildly increased FDP, and markedly increased total PAI-1 [25, 26]. Patients with hypomagnesemia had more activated coagulation status than those with normal magnesium level or hypermagnesemia and it was significantly associated with DIC after adjusting for several confounders, including APACHE II score (effect of severity), serum lactate levels (effect of microcirculation) [27], and bilirubin levels (effect of hepatic inflammatory and coagulation processes) [28]. Therefore, hypomagnesemia was associated with the type of DIC that was typical of sepsis, and might have deteriorated coagulopathy caused by sepsis.

Although there have been no previous studies on the association between serum magnesium concentration and coagulopathy, our hypothesis that hypomagnesemia is associated with coagulopathy in sepsis might be biologically plausible based on the association of hypomagnesemia with high-mobility group box 1 (HMGB1), a damage-associated molecular pattern and a lethal mediator of sepsis [29] and inflammatory cytokines [13]. Experimental studies have also shown that magnesium deficiency promotes the secretion of HMGB1 proteins from lipopolysaccharide-activated macrophages in vitro [14], magnesium sulfate reduces serum HMGB1 levels in cecal ligation and puncture rat models [30], and HMGB1 in the systemic circulation promotes the development of DIC in rats [31]. Magnesium deficiency in a rat model of endotoxin shock also promoted the secretion of proinflammatory cytokines, including interleukin-6, tumor necrosis factor-alpha, and interleukin-1-beta [5], which intensify the failure of various organs [9, 11]. These cytokines activate coagulation via crosstalk between inflammation and coagulation [15].

We also investigated whether hypomagnesemia on day 3 or day 5 was associated with coagulation parameters. Hypomagnesemia on ICU admission and day 3 was associated with activated coagulation and suppressed fibrinolysis status. Time course of coagulation parameters from admission to day 3 according to serum magnesium status showed there was no relation between serum magnesium levels and coagulation parameters among patients with hypomagnesemia on ICU admission, whether hypomagnesemia had resolved or persisted. However, newly developed hypomagnesemia on day 3 was associated with activated coagulation and suppressed fibrinolysis status. Thus, magnesium supplementation for hypomagnesemia or for hypomagnesemia prophylaxis, especially from ICU admission to day 3, might be a potential therapeutic strategy for the treatment of DIC in sepsis though we did not investigate the influence of magnesium administration and anticoagulation therapy for DIC on the results.

Interestingly, hypomagnesemia on ICU admission in patients with abdominal sepsis was not associated with DIC. Patients with abdominal sepsis had lower severity (APACHE II score (median: 22 vs 26; *P* < 0.001), lower hospital mortality (11.9% vs. 22.9%; *P* < 0.001), and more frequent emergency surgery to control the source of infection (74.9% vs. 22.6%; *P* < 0.001) than those with non-abdominal sepsis. Severity and source control management might be involved in difference of the association with DIC between abdominal sepsis and non-abdominal sepsis. We are considering a future prospective study to investigate the association between magnesium and coagulation, including the measurement of cytokines and mediators, to further confirm our hypothesis.

Magnesium deficiency has been implicated in electrolyte disturbances such as hypocalcemia [1, 3]. Ionized calcium (factor IV) anchors the side chains of coagulation factors to the phospholipids of the platelet membrane and plays an important role in the conversion of fibrinogen to fibrin [32]. Decreased ionized calcium is common in sepsis [33]. It is hypothesized that sepsis causes the impaired secretion of parathyroid hormone, increases the organ resistance to parathyroid hormone or promotes influx from the blood to tissues [33–35]. However, it is not known whether hypocalcemia impairs coagulation function or sepsis-induced coagulopathy causes hypocalcemia as the result of serum calcium consumption [32]. Although the exact mechanism of magnesium-calcium interaction is unknown, hypomagnesemia generally impairs the secretion of parathyroid hormone or increases resistance to parathyroid hormone, leading to hypocalcemia [36]. Additionally, we investigated the association between serum ionized calcium and coagulation. Our study showed that patients with hypomagnesemia had low ionized calcium concentrations (Table 1) and 574 patients (76.2%) had hypocalcemia (normal range: 1.12 to 1.32 mmol/L) and low ionized calcium concentrations that tended to be associated with abnormal coagulation function (Additional file 11). Thus, the effects of magnesium on coagulopathy may be mediated partly through calcium, as well as through inflammation. Additionally, a previous study reported that serum zinc level was associated with sepsis-induced coagulopathy [37]. Magnesium and zinc might have deep crosstalk. Further research is required to clarify the mechanism of magnesium on coagulation function with a particular focus on the interaction between magnesium, calcium, and between magnesium and trace element.

Our study had several limitations. First, this was a single-center, observational, retrospective study; thus, there is a possibility of selection bias or unmeasured confounding factors due to missing data. Second, we did not have information about the magnesium administration during ICU stay, and the baseline magnesium profile before ICU admission, such as magnesium deficiency or overload, or other factors affecting magnesium status, such as drugs (diuretics, antibiotics, chemotherapy, proton-pump inhibitors, laxatives, etc.), comorbidities (gastrointestinal disorder, nutritional disorder), and magnesium supplementation [3]. Third, we did not find an association between magnesium deficiency and coagulopathy, but only an association between hypomagnesemia and coagulopathy. Hypomagnesemia, defined by low levels of serum ionized magnesium (a physiologically active form of magnesium in the plasma), is not necessarily equal to magnesium deficiency because intracellular magnesium primarily functions as a cofactor for biochemical and physiological processes [38, 39]. Finally, we did not demonstrate activate coagulation via crosstalk between inflammation and coagulation on which our hypothesis was based.

## Conclusion

To our knowledge, this is the first study to describe the association between serum magnesium concentration and coagulopathy in sepsis. In this study, we showed that patients with hypomagnesemia had a significantly activated coagulation status, such as suppressed-fibrinolytic DIC due to sepsis. Hypomagnesemia may promote inflammation and intensify coagulopathy due to the crosstalk between inflammation and coagulation. Therefore, the treatment of hypomagnesemia may be a potential therapeutic strategy for the treatment of coagulopathy in sepsis. Further studies are required to clarify the mechanism of the relationship between serum magnesium and coagulation and whether treatment of hypomagnesemia improves coagulopathy.

## Supplementary Information

**Additional file 1:** Patient characteristics and laboratory parameters of the study population by DIC status

**Additional file 2:** Histogram of serum ionized calcium concentration by DIC status.

**Additional file 3:** Coagulation parameters for each serum magnesium level.

**Additional file 4:** Coagulation parameters between different serum magnesium levels (hypomagnesemia, normal magnesium level, and hypermagnesemia) on day 3 in sepsis.

**Additional file 5:** Coagulation parameters between different serum magnesium levels (hypomagnesemia, normal magnesium level, and hypermagnesemia) on day 5 in sepsis.

**Additional file 6:** Time course of coagulation parameters in persistent hypomagnesemia and resolved hypomagnesemia on day 3.

**Additional file 7:**Time course of coagulation parameters in developed hypomagnesemia and non-hypomagnesemia on day 3.

**Additional file 8:** Receiver operating characteristic curves of serum magnesium concentration for DIC.

**Additional file 9:** Logistic regression analyses of the disseminated intravascular coagulation in patients with abdominal sepsis.

**Additional file 10:** Logistic regression analyses of the disseminated intravascular coagulation in patients with non-abdominal sepsis.

**Additional file 11:** Coagulation parameters according to the quartiles of serum ionized calcium.

## Data Availability

The datasets generated and/or analyzed during the current study are not publicly available because of patient-related confidentiality, but are available from the corresponding author upon reasonable request.

## Abbreviations

AT: Antithrombin
APACHE: Acute Physiology and Chronic Health Evaluation
BMI: Body mass index
CRP: C-reactive protein
DIC: Disseminated intravascular coagulation
FDP: Fibrin degradation products
HMGB1: High-mobility group box 1
ICU: Intensive care unit
ISTH: International Society on Thrombosis and Haemostasis
PAI-1: Plasminogen activator inhibitor-1
PC: Protein C
PIC: Plasmin-α2 plasmin inhibitor complex
PT-INR: Prothrombin time-international normalized ratio
TAT: Thrombin-antithrombin complex
