# Supplementary material for: Association between hypomagnesemia and coagulopathy in sepsis: a retrospective observational study
Source: BMC Anesthesiol. 2022 Nov 24;22:359. doi: 10.1186/s12871-022-01903-2 (PMC9685885; doi:10.1186/s12871-022-01903-2)
Supplement: Supplementary file 1 — Additional file 1: Patient characteristics and laboratory parameters of the study population by DIC status [file 12871_2022_1903_MOESM1_ESM.docx]

**Additional file 1**

Patient characteristics and laboratory parameters of the study population by DIC status.

|  | DIC (N = 181) | Non–DIC (N = 572) | *P*–value |
| --- | --- | --- | --- |
| Age, y, median (IQR) | 69 (61–78) | 68 (58–77) | 0.30 |
| Male, n (%) | 102 (56.4) | 314 (54.9) | 0.73 |
| BMI | 22.7 (19.6–24.9) | 22.1(19.5–25.5) | 0.78 |
| Origin of sepsis, n (%) |  |  | 0.015 |
| Abdomen | 89 (49.2) | 306(53.5) |  |
| Thorax | 31 (17.1) | 130 (22.7) |  |
| Urinary tract | 14 (7.7) | 19 (3.3) |  |
| Other | 47 (25.0) | 117 (20.5) |  |
| Medical history, n (%) |  |  |  |
| Anticoagulation therapy | 6 (3.3) | 50 (89.3) | 0.015 |
| CKD with dialysis | 11 (6.1) | 45 (7.9) | 0.42 |
| Diabetes mellitus | 40 (22.1) | 170 (29.7) | 0.046 |
| Chronic liver disease | 23 (12.7) | 47 (8.2) | 0.070 |
| Hypertension | 89 (49.2) | 290 (50.7) | 0.72 |
| Laboratory values, median (IQR) |  |  |  |
| Magnesium, mg/dL | 1.9 (1.6–2.2) | 2.0 (1.8–2.2) | 0.007 |
| Albumin, mg/dL | 2.4 (2.0–2.8) | 2.4 (2.0–2.80) | 0.66 |
| Bilirubin, mg/dL | 1.09 (0.66–2.07) | 0.81 (0.56–1.30) | < 0.001 |
| Creatinine, mg/dL | 1.82 (1.07–2.85) | 0.96 (0.66–1.88) | < 0.001 |
| CRP, mg/dL | 13.0 (6.1–23.3) | 12.7 (5.6–22.7) | 0.41 |
| Ionized calcium, mmol/L | 1.01(0.95–1.07) | 1.06(1.0–1.12) | < 0.001 |
| Lactate, mmol/L | 3.8 (2.2–7.1) | 2.0 (1.3–3.1) | < 0.001 |
| Coagulation values, median (IQR) |  |  |  |
| Platelet, 10^4^/μL | 7.0 (4.4–10.3) | 16.6 (12.2–23.9) | < 0.001 |
| FDP, μg/mL | 35.9 (22.1–67.6) | 14.5 (9.3–21.8) | < 0.001 |
| PT – INR | 1.68 (1.52–2.03) | 1.31 (1.21–1.46) | < 0.001 |
| Fibrinogen, mg/dL | 270 (154–375) | 370 (255–511) | < 0.001 |
| Protein C activity, % | 33.6 (25.5–42.5) | 57.5 (43.8–74.1) | < 0.001 |
| Antithrombin III activity, % | 40.9 (30.9–52.5) | 57.3 (45.9–72.0) | < 0.001 |
| TAT, ng/mL | 17.2 (10.4–35.4) | 9.4 (5.7–16.8) | < 0.001 |
| PIC, μg/mL | 1.5 (0.8–3.1) | 1.3 (0.8–1.9) | 0.006 |
| PAI–1, ng/mL | 242 (97.0–762.5) | 81.0 (40.0–218.8) | < 0.001 |
| Organ support, n (%) |  |  |  |
| Mechanical ventilation | 157 (86.7) | 468 (81.8) | 0.12 |
| Renal replacement therapy | 83 (45.9) | 98 (17.1) | < 0.001 |
| Severity of disease |  |  |  |
| SOFA score on ICU admission, median (IQR) | 10 (8–12) | 6 (4–8) | < 0.001 |
| APACHE Ⅱ score, median (IQR) | 29 (22–34) | 23 (17–27) | < 0.001 |
| PaO_2_/F_I_O_2_ ratio, median (IQR) | 231 (152–319) | 268 (173–370) | 0.001 |
| Septic shock, n (%) | 136 (75.1) | 215 (37.7) | < 0.001 |
| Hospital mortality, n (%) | 61 (33.7) | 68 (11.9) | < 0.001 |

Continuous variables are presented as medians with interquartile ranges (first to third quartiles). Categorical variables are presented as counts and percentiles.

Definitions of abbreviations: DIC, disseminated intravascular coagulation; IQR, interquartile range (first quartile to third quartile); BMI, body mass index; CKD, chronic kidney disease; CRP, C-reactive protein; FDP, fibrin degradation products; PT-INR, prothrombin time-international normalized ratio; TAT, thrombin-antithrombin complex; PIC, plasmin-α2 plasmin inhibitor complex; PAI-1, plasminogen activator inhibitor-1; SOFA, Sequential Organ Failure Assessment; APACHE, Acute Physiology and Chronic Health Evaluation; PaO_2_, partial pressure of oxygen; F_I_O_2_, fraction of inspired oxygen.
