# Supplementary material for: Association between hypomagnesemia and coagulopathy in sepsis: a retrospective observational study
Source: BMC Anesthesiol. 2022 Nov 24;22:359. doi: 10.1186/s12871-022-01903-2 (PMC9685885; doi:10.1186/s12871-022-01903-2)
Supplement: Supplementary file 2 — Additional file 2: Histogram of serum ionized calcium concentration by DIC status. [file 12871_2022_1903_MOESM2_ESM.docx]

**Additional file 2**


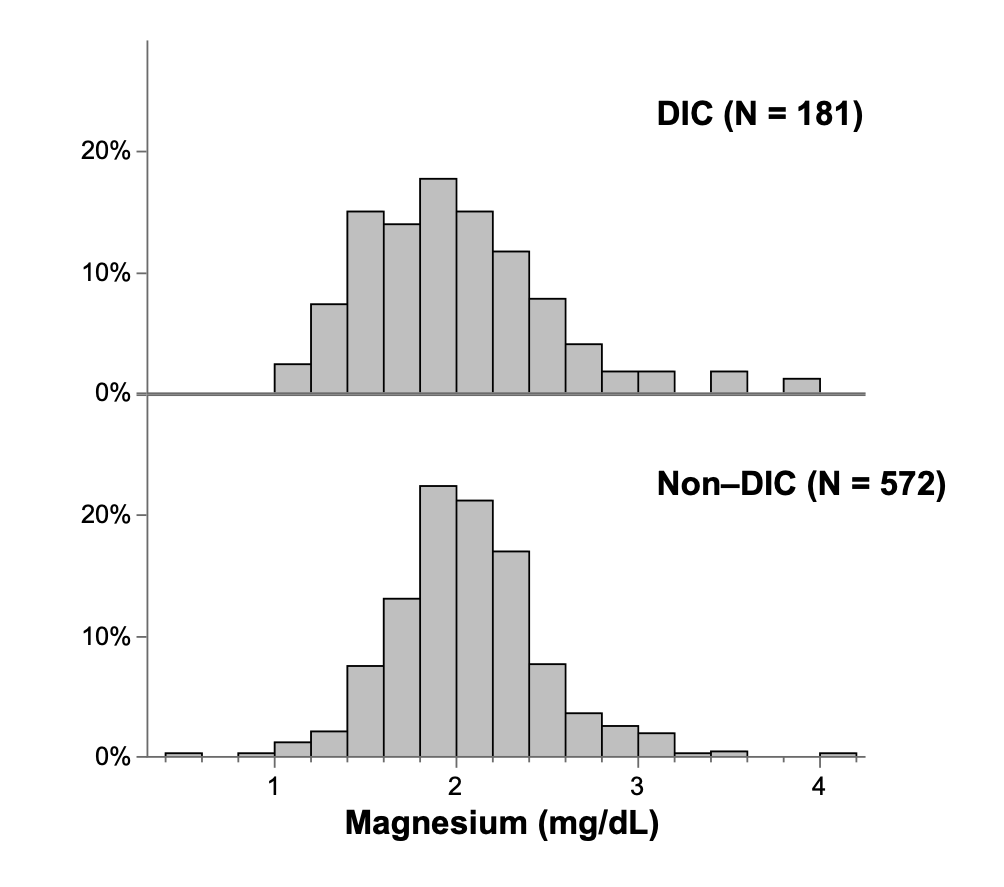

**Histogram of serum magnesium concentration by DIC status.**According to the ISTH criteria, 181 and 572 patients were categorized as DIC and non-DIC respectively. y-axis is plotted in relative frequency. Abbreviations: ISTH, International Society on Thrombosis and Hemostasis; DIC, disseminated intravascular coagulation.


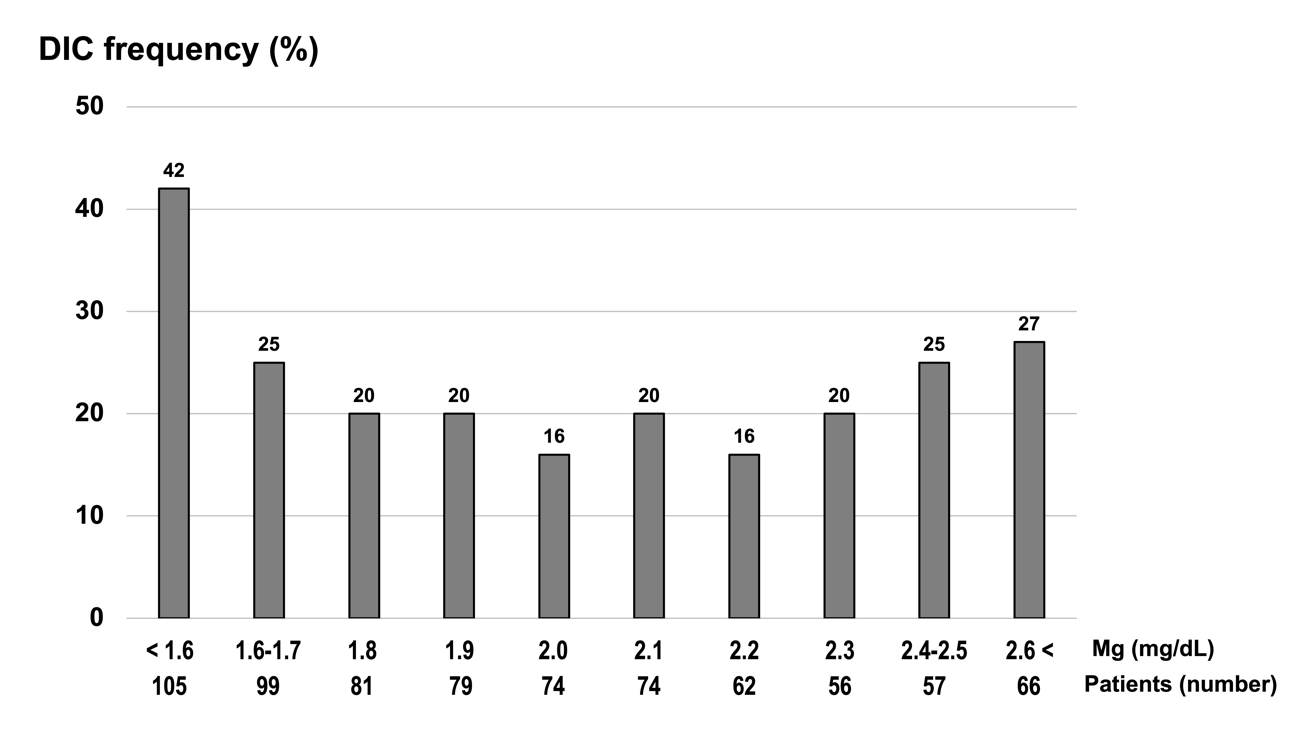


**The frequency of DIC according to decile of serum magnesium concentration.**The frequency of DIC were sorted into ten equal parts according to serum magnesium concentration on ICU admission. Abbreviations: DIC, disseminated intravascular coagulation; Mg, magnesium.
