# Supplementary material for: Association between hypomagnesemia and coagulopathy in sepsis: a retrospective observational study
Source: BMC Anesthesiol. 2022 Nov 24;22:359. doi: 10.1186/s12871-022-01903-2 (PMC9685885; doi:10.1186/s12871-022-01903-2)
Supplement: Supplementary file 3 — Additional file 3: Coagulation parameters for each serum magnesium level. [file 12871_2022_1903_MOESM3_ESM.docx]

**Additional file 3**

Coagulation parameters for each serum magnesium level.

|  | Hypomagnesemia  (< 1.6 mg/dL) N = 105 | Normal Mg level  (1.6–2.4 mg/dL) N = 552 | Hypermagnesemia  (> 2.4 mg/dL) N = 96 | *P*–value |
| --- | --- | --- | --- | --- |
| Coagulation values, median (IQR) |  |  |  |  |
| Platelet, 10^4^/μL | 11.2 (5.5–16.6) | 14.9 (9.6–22.8) * | 15.1 (10.0–19.9) * | < 0.001 |
| FDP, μg/mL | 18.9 (12.5–41.9) | 16.6 (9.90–27.7) | 16.8 (12.4–29.8) | 0.061 |
| PT – INR | 1.52 (1.31–1.82) | 1.37 (1.23–1.55) * | 1.36 (1.23–1.61) * | < 0.001 |
| Fibrinogen, mg/dL | 268 (175–347) | 356 (245–501) * | 371 (222–525) * | < 0.001 |
| Protein C activity, % | 42.6 (32.4–55.6) | 52.5 (38.6–70.0) * | 53.4 (36.5–71.7) * | 0.001 |
| Antithrombin III activity, % | 46.2 (35.6–55.3) | 55.1 (43.0–69.1) * | 57.3 (42.1–68.5) * | < 0.001 |
| TAT, ng/mL | 16.1(8.4–28.3) | 10.0 (5.8–18.7) * | 11.1 (6.0–20.4) * | < 0.001 |
| PIC, μg/mL | 1.4 (0.7–2.2) | 1.3 (0.8–2.1) | 1.2 (0.8–1.9) | 0.64 |
| PAI–1, ng/mL | 242.0 (111.2–661.0) | 97.0 (45.8–221.0) * | 83.0 (48.0–334.0) | 0.001 |

Continuous variables are presented as medians with interquartile ranges (first to third quartiles). Categorical variables are presented as counts and percentiles.

Abbreviations: Mg, magnesium; IQR, interquartile range (first quartile to third quartile); FDP, fibrin degradation products; PT-INR, prothrombin time-international normalized ratio; TAT, thrombin-antithrombin complex; PIC, plasmin-α2 plasmin inhibitor complex; PAI-1, plasminogen activator inhibitor-1.

**P* value < 0.05, comparison versus hypomagnesemia (Steel–Dwass test or the chi-square test with Bonferroni correction).

†*P* value < 0.05, comparison versus normal level (Steel–Dwass test or the chi-square test with Bonferroni correction).
