# Supplementary material for: Association between hypomagnesemia and coagulopathy in sepsis: a retrospective observational study
Source: BMC Anesthesiol. 2022 Nov 24;22:359. doi: 10.1186/s12871-022-01903-2 (PMC9685885; doi:10.1186/s12871-022-01903-2)
Supplement: Supplementary file 5 — Additional file 5: Coagulation parameters between different serum magnesium levels (hypomagnesemia, normal magnesium level, and hypermagnesemia) on day 5 in sepsis. [file 12871_2022_1903_MOESM5_ESM.docx]

**Additional file 5**


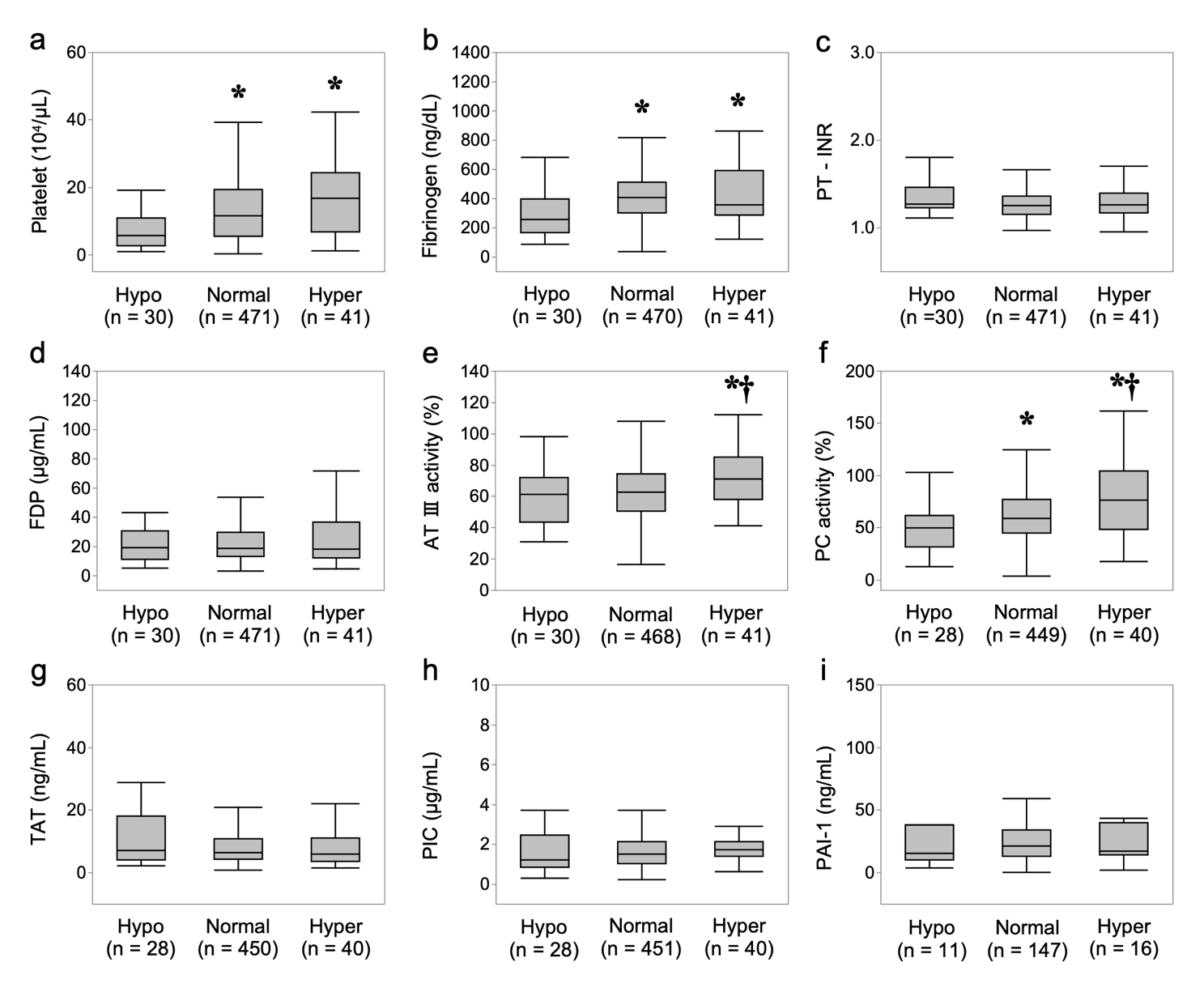


**Coagulation parameters between different serum magnesium levels (hypomagnesemia, normal magnesium level, and hypermagnesemia) on day 5 in sepsis.**
Box-and-whisker plot depicting a difference in coagulation parameters between different serum magnesium levels on day 3 (hypomagnesemia [Hypo] vs. normal magnesium level [Normal] vs. hypermagnesemia [Hyper]) in patients with sepsis admitted to the ICU. Admission serum levels of **(a)** platelet count (10^4^/μL); **(b)** fibrinogen (mg/dL); **(c)** PT-INR; **(d)** FDP (μg/mL); **(e)** AT Ⅲ activity (%); **(f)** PC activity (%); **(g)** TAT (ng/mL); **(h)** PIC (μg/mL); **(i)** PAI-1 (ng/mL). Boxplots display median with first and third quartile, and whiskers indicate smallest and largest nonoutlier observations. **P*-value < 0.05, comparison versus hypomagnesemia (Steel–Dwass test). †*P*-value < 0.05, comparison versus normal level (Steel–Dwass test). Abbreviations: AT III, antithrombin III; FDP, fibrin degradation products; ICU, intensive care unit; PC, protein C; PIC, plasmin-α2 plasmin inhibitor complex; PT-INR, prothrombin time-international normalized ratio; TAT, thrombin-antithrombin complex; PAI-1, plasminogen activator inhibitor-1.
