# Supplementary material for: Association between hypomagnesemia and coagulopathy in sepsis: a retrospective observational study
Source: BMC Anesthesiol. 2022 Nov 24;22:359. doi: 10.1186/s12871-022-01903-2 (PMC9685885; doi:10.1186/s12871-022-01903-2)
Supplement: Supplementary file 6 — Additional file 6: Time course of coagulation parameters in persistent hypomagnesemia and resolved hypomagnesemia on day 3. [file 12871_2022_1903_MOESM6_ESM.docx]

**Additional file 6**


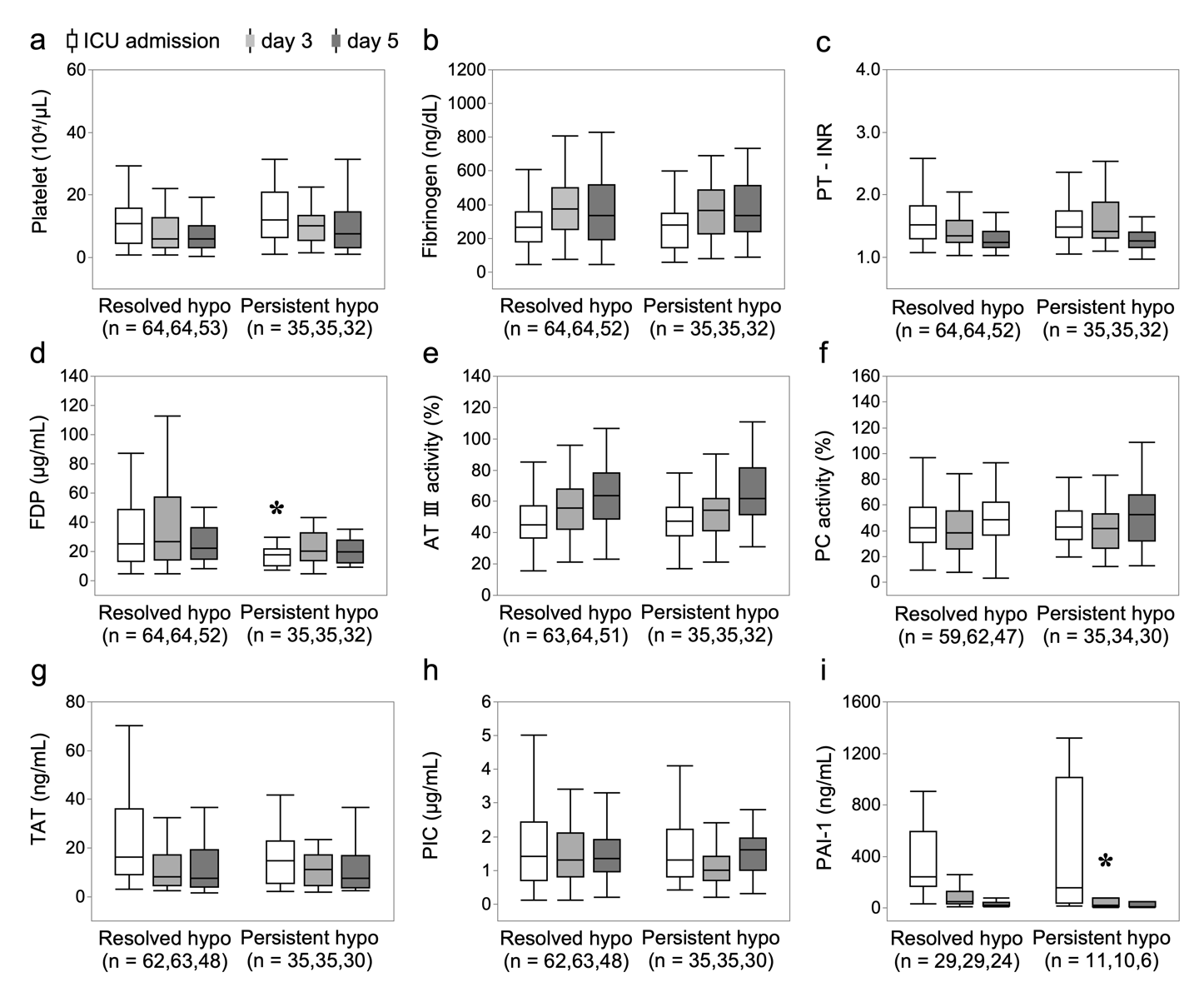


**Time course of coagulation parameters in persistent hypomagnesemia and resolved hypomagnesemia on day 3.**
Among 105 patients with hypomagnesemia (Mg < 1.6 mg/dL) on admission day, 99 patients were categorized into persistent hypomagnesemia group (Mg < 1.6 mg/dL on day 3) (N = 35) and resolved hypomagnesemia group (Mg ≧ 1.6 mg/dL on day 3) (N = 64) according to the serum magnesium concentration on day 3. Box-and-whisker plot depicting a difference in coagulation parameters between persistent hypomagnesemia and resolved hypomagnesemia. Admission serum levels of **(a)** platelet count (10^4^/μL); **(b)** fibrinogen (mg/dL); **(c)** PT-INR; **(d)** FDP (μg/mL); **(e)** AT Ⅲ activity (%); **(f)** PC activity (%); **(g)** TAT (ng/mL); **(h)** PIC (μg/mL); **(i)** PAI-1 (ng/mL). Boxplots display median with first and third quartile, and whiskers indicate smallest and largest nonoutlier observations. **P*-value < 0.05, comparison between resolved hypomagnesemia and persistent hypomagnesemia at the same time (Mann–Whitney *U* test). Abbreviations: hypo, hypomagnesemia; AT III, antithrombin III; FDP, fibrin degradation products; ICU, intensive care unit; PC, protein C; PIC, plasmin-α2 plasmin inhibitor complex; PT-INR, prothrombin time-international normalized ratio; TAT, thrombin-antithrombin complex; PAI-1, plasminogen activator inhibitor-1.
