# Supplementary material for: Association between hypomagnesemia and coagulopathy in sepsis: a retrospective observational study
Source: BMC Anesthesiol. 2022 Nov 24;22:359. doi: 10.1186/s12871-022-01903-2 (PMC9685885; doi:10.1186/s12871-022-01903-2)
Supplement: Supplementary file 8 — Additional file 8: Receiver operating characteristic curves of serum magnesium concentration for DIC. [file 12871_2022_1903_MOESM8_ESM.docx]

**Additional file 8**


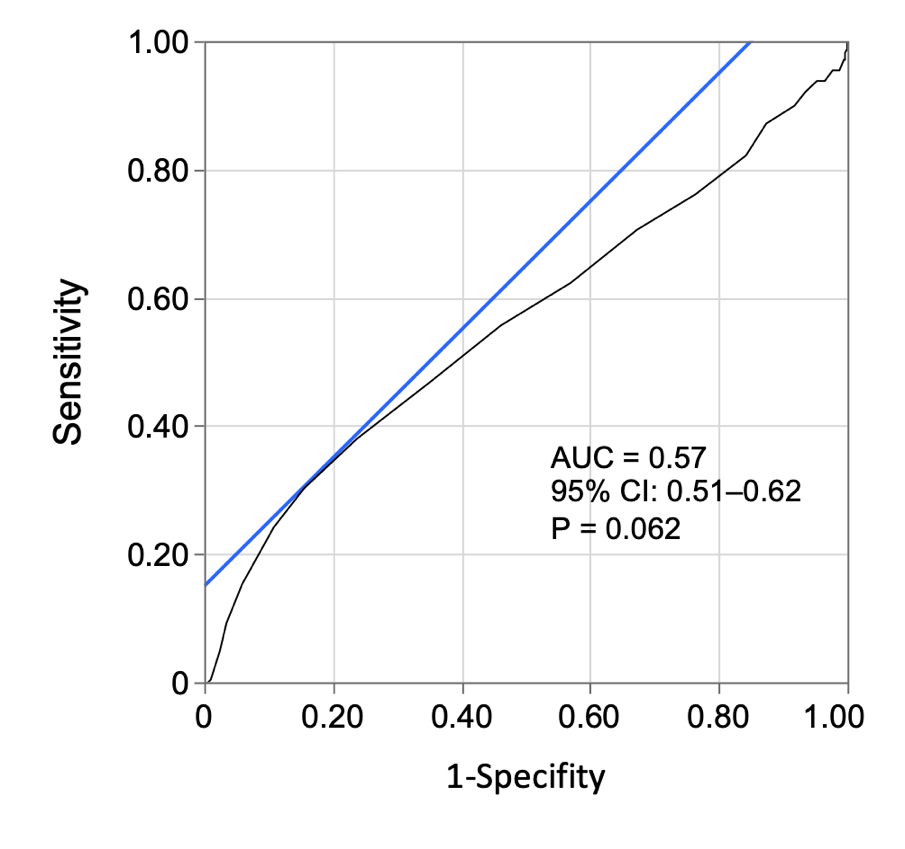

**Receiver operating characteristic curve of serum magnesium concentration for DIC.**Serum magnesium concentration showed sensitivity 30.4%, specificity 84.6%, a cut-off of 1.6mg/dL, and AUC = 0.57 (95% CI 0.51–0.62). Abbreviations: DIC, disseminated intravascular coagulation; AUC, areas under the curves; CI, confidence intervals.
