# Supplementary material for: Association between hypomagnesemia and coagulopathy in sepsis: a retrospective observational study
Source: BMC Anesthesiol. 2022 Nov 24;22:359. doi: 10.1186/s12871-022-01903-2 (PMC9685885; doi:10.1186/s12871-022-01903-2)
Supplement: Supplementary file 9 — Additional file 9: Logistic regression analyses of the disseminated intravascular coagulation in patients with abdominal sepsis. [file 12871_2022_1903_MOESM9_ESM.docx]

**Additional file 9**

Logistic regression analyses of the disseminated intravascular coagulation in patients with abdominal sepsis.

|  | Univariate logistic regression  N = 395 | | | Multivariate logistic regression  N = 395 | | |
| --- | --- | --- | --- | --- | --- | --- |
|  | OR | 95% CI | P-value | OR | 95% CI | *P-*value |
| Magnesium |  | | | | | |
| Normal Mg level | Reference |  | | Reference |  | |
| Hypomagnesemia | 1.70 | 0.86–3.38 | 0.13 | 0.88 | 0.39–2.02 | 0.77 |
| Hypermagnesemia | 1.61 | 0.78–3.33 | 0.20 | 0.94 | 0.38–2.28 | 0.89 |
| Male | 0.97 | 0.60–1.55 | 0.90 | 0.89 | 0.52–1.53 | 0.68 |
| APACHE Ⅱ score, per score | 1.09 | 1.06–1.13 | < 0.001 | 1.03 | 0.99–1.08 | 0.11 |
| Bilirubin, per mg/dL | 1.09 | 1.01–1.17 | 0.025 | 1.06 | 0.97–1.15 | 0.22 |
| Creatinine, per mg/dL | 1.15 | 1.03–1.26 | 0.013 | 1.08 | 0.94–1.24 | 0.29 |
| CRP, per mg/dL | 1.01 | 0.99–1.04 | 0.23 | 1.02 | 0.99–1.05 | 0.11 |
| Ionized calcium, per mmol/L | 0.011 | 0.001–0.15 | 0.001 | 0.12 | 0.006–2.65 | 0.18 |
| Lactate, per mmol/L | 1.46 | 1.30–1.64 | < 0.001 | 1.43 | 1.25–1.64 | < 0.001 |

Logistic regression analyses for disseminated intravascular coagulation.

Data are expressed as odds ratios (95% CI). Logistic regression analyses were performed for the complete-case analysis. A total of 753 participants were included in the multivariate logistic regression analyses. Abbreviations: OR, odds ratio; CI, confidence interval; Mg, magnesium; APACHE, Acute Physiology and Chronic Health Evaluation; CRP, C-reactive protein
