# Supplementary material for: Association between hypomagnesemia and coagulopathy in sepsis: a retrospective observational study
Source: BMC Anesthesiol. 2022 Nov 24;22:359. doi: 10.1186/s12871-022-01903-2 (PMC9685885; doi:10.1186/s12871-022-01903-2)
Supplement: Supplementary file 10 — Additional file 10: Logistic regression analyses of the disseminated intravascular coagulation in patients with non-abdominal sepsis. [file 12871_2022_1903_MOESM10_ESM.docx]

|  | Univariate logistic regression  N = 358 | | | Multivariate logistic regression  N = 358 | | |
| --- | --- | --- | --- | --- | --- | --- |
|  | OR | 95% CI | *P*-value | OR | 95% CI | *P-*value |
| Magnesium |  | | | | | |
| Normal Mg level | Reference |  | | Reference |  | |
| Hypomagnesemia | 3.91 | 2.16–7.12 | < 0.001 | 3.04 | 1.46–6.32 | 0.003 |
| Hypermagnesemia | 0.95 | 0.45–1.96 | 0.88 | 0.74 | 0.31–1.77 | 0.50 |
| Male | 1.15 | 0.71–1.86 | 0.57 | 1.21 | 0.67–2.17 | 0.53 |
| APACHE Ⅱ score, per score | 1.12 | 1.08–1.17 | < 0.001 | 1.10 | 1.06–1.14 | < 0.001 |
| Bilirubin, per mg/dL | 1.30 | 1.10–1.54 | < 0.001 | 1.26 | 1.05–1.53 | 0.007 |
| Creatinine, per mg/dL | 1.12 | 1.02–1.24 | 0.023 | 1.04 | 0.91–1.19 | 0.58 |
| CRP, per mg/dL | 1.00 | 0.97–1.02 | 0.79 | 1.01 | 0.99–1.05 | 0.32 |
| Ionized calcium, per mmol/L | 0.005 | 0.001–0.054 | < 0.001 | 0.093 | 0.005–1.49 | 0.094 |
| Lactate, per mmol/L | 1.32 | 1.21–1.44 | < 0.001 | 1.14 | 1.04–1.25 | 0.003 |

**Additional file 10**

Logistic regression analyses of the disseminated intravascular coagulation in patients with non-abdominal sepsis.

Logistic regression analyses for disseminated intravascular coagulation.

Data are expressed as odds ratios (95% CI). Logistic regression analyses were performed for the complete-case analysis. A total of 753 participants were included in the multivariate logistic regression analyses. Abbreviations: OR, odds ratio; CI, confidence interval; Mg, magnesium; APACHE, Acute Physiology and Chronic Health Evaluation; CRP, C-reactive protein
