# Supplementary material for: Association between hypomagnesemia and coagulopathy in sepsis: a retrospective observational study
Source: BMC Anesthesiol. 2022 Nov 24;22:359. doi: 10.1186/s12871-022-01903-2 (PMC9685885; doi:10.1186/s12871-022-01903-2)
Supplement: Supplementary file 11 — Additional file 11: Coagulation parameters according to the quartiles of serum ionized calcium. [file 12871_2022_1903_MOESM11_ESM.docx]

**Additional file 11**

Coagulation parameters according to the quartiles of serum ionized calcium.

| Ca^++^ quartile, mmol/L | First quartile  (<0.99)  N = 201 | Second quartile  (0.99–1.05)  N = 187 | Third quartile  (1.06–1.11)  N = 186 | Forth quartile  (>1.11)  N = 179 | *P*–value |
| --- | --- | --- | --- | --- | --- |
| Coagulation values, median (IQR) | | | | | |
| Platelet, 10^4^/μL | 11.5 (6.5– 18.5) | 14.9 (9.7–20.9) | 15.4 (10.3– 23.6) | 16.1 (11.7– 22.9) | < 0.001 |
| FDP, μg/mL | 19.3 (11.7–34.4) | 19.9 (11.0–34.5) | 17.1 (10.5– 27.1) | 14.7 (9.6– 22.3) | 0.003 |
| PT – INR | 1.48 (1.31–1.76) | 1.39 (1.27–1.58) | 1.35 (1.23–1.54) | 1.30 (1.20– 1.46) | < 0.001 |
| Fibrinogen, mg/dL | 298 (200–437) | 342 (242–467) | 343 (240–486) | 399 (270–559) | < 0.001 |
| Protein C activity, % | 40.3 (29.9–54.9) | 50.3 (38.7–63.6) | 52.7(39.0–71.3) | 63.8(46.4– 81.6) | < 0.001 |
| Antithrombin III activity, % | 47.2 (35.2–57.9) | 51.7 (39.9–60.8) | 56.2 (46.4– 71.5) | 63.3 (48.7– 76.8) | < 0.001 |
| TAT, ng/mL | 11.6 (5.9–21.7) | 10.8 (6.2–19.8) | 10.2 (6.6–18.9) | 9.6 (5.9–19.7) | 0.58 |
| PIC, μg/mL | 1.2 (0.60–2.0) | 1.2 (0.8–2.0) | 1.4 (0.9–2.5) | 1.5 (0.9–2.1) | 0.007 |
| PAI–1, ng/mL | 142 (65–270) | 126(58–262) | 84 (34–237) | 75 (44–184) | 0.01 |
| ISTH score, median (IQR) | 4 (3–5) | 4(2–5) | 3 (2–4) | 3 (2–4) | < 0.001 |
| DIC, n (%) | 68 (33.8) | 51 (27.3) | 40 (21.5) | 22 (12.3) | < 0.001 |

Continuous variables are presented as medians with interquartile ranges (first to third quartiles). Categorical variables are presented as counts and percentiles.

Abbreviations: Ca^++^, ionized calcium, IQR, interquartile range (first quartile to third quartile); FDP, fibrin degradation products; PT-INR, prothrombin time-international normalized ratio; TAT, thrombin-antithrombin complex; PIC, plasmin-α2 plasmin inhibitor complex; PAI-1, plasminogen activator inhibitor-1; ISTH, International Society on Thrombosis and Hemostasis; DIC, disseminated intravascular coagulation.
